# A novel adenosine-to-inosine RNA editing-based nomogram for predicting prognosis of hepatocellular carcinoma

**DOI:** 10.3389/fphar.2025.1547320

**Published:** 2025-05-14

**Authors:** Shiqiong Huang, Ji Sun

**Affiliations:** Department of Pharmacy, The First Hospital of Changsha, Changsha, China

**Keywords:** adenosine-to-inosine RNA editing, hepatocellular carcinoma, risk score, prognosis, host genes

## Abstract

**Objective:**

Although the role of adenosine-to-inosine RNA editing (ATIRE) has gained widespread attention in multiple cancers, its predictive role in hepatocellular carcinoma (HCC) remains little known. We aimed to establish a predicting signature based on ATIRE for the prognosis of HCC.

**Methods:**

A total of 200 HCC patients with survival data and ATIRE profiles from The Cancer Genome Atlas (TCGA) database were divided into training (n = 140) and validation (n = 60) cohorts. Survival-related ATIRE sites were identified by the least absolute shrinkage and selection operator algorithm. ATIRE-based risk score was then generated with these ATIRE sites. Cox proportional hazards regression was employed to construct the ATIRE-based nomogram signature. The receiver operating characteristic (ROC) curve was used to evaluate the predictive efficacy of the signature. Harrell’s C-index and calibration plot was utilized to evaluate the significant prognostic factors.

**Results:**

Nine ATIRE sites were screened to establish the ATIRE risk score, and it was found to be associated with prognosis of HCC. Survival analysis revealed that higher ATIRE-based risk scores were significantly associated with worse overall survival (OS) in both the training dataset (*p* < 0.001) and the validation dataset (*p* = 0.011), as well as in the combined dataset (*p* < 0.001). The ROC curve displayed a good predictive efficiency of the risk score regarding 1-year, 2-year, and 3-year OS. Furthermore, ATIRE sites were significantly correlated with the expression of host genes and were likely to be involved in certain cancer-related pathways.

**Discussion:**

Our findings provided a novel ATIRE-based nomogram, which could serve as a potential tool for predicting HCC prognosis.

## 1 Introduction

Hepatocellular carcinoma (HCC), accounting for 75%–85% of primary liver cancer, is one of the most common malignant tumors and the leading cause of cancer-related deaths worldwide (Yang et al., 2019; Vogel et al., 2022). HCC has a chronic disease course and various etiologies, with a 5-year survival rate of approximately 18% (Vogel et al., 2022; Villanueva, 2019). Due to the similarity between the incidence and mortality (both approximately 830,000 deaths per year), HCC has a dismal prognosis (Siegel et al., 2022). Therefore, novel promising prognostic markers or models are needed to improve the survival rate of HCC patients.

RNA editing is a posttranscriptional process that confers RNA sequence changes in a site-specific manner (Gott and Emeson, 2000; Christofi and Zaravinos, 2019). The most common type of RNA editing is adenosine-to-inosine RNA editing (ATIRE) (Eisenberg and Levanon, 2018) catalyzed by double-stranded RNA-specific adenosine deaminase acting on RNA (ADAR) enzymes. Intriguingly, ATIRE was found to be involved in tumorigenesis by participating in several biological processes (Baysal et al., 2017). Meanwhile, ATIRE also had impacts on the progression (Han et al., 2020), metastasis (Fu et al., 2017), and drug resistance (Wood et al., 2021) of cancer.

ATIRE sites have been extensively detected in the human genome (Bazak et al., 2014). A number of ATIRE sites were identified to be correlated with clinical outcomes in patients with cancer (Han et al., 2015). Specifically, there was strong evidence that ATIRE played a vital role in the biological processes of HCC. Chen et al. (2013) performed an integrative RNA-Seq analysis on HCC tissue specimens and identified an ATIRE event involved in HCC progression. They also revealed that ADAR1-mediated antizyme inhibitor 1 (*AZIN1*) RNA editing was associated with tumor initiation and development in HCC. Another genome-wide study of human RNA editing reported that RNA editing of bladder cancer-associated protein (*BLCAP*) might promote HCC cell proliferation through activating the AKT/mTOR signal pathway (Hu et al., 2015).

Notably, transcriptome-wide ATIRE profiling was performed in cancer by recent bioinformatics analyses (Liu et al., 2022), which might underline the potential application of ATIRE as a cancer biomarker. However, the correlation of ATIRE sites and their prognostic value remains little known in HCC, and there is a shortage of prognostic prediction models related to RNA editing in HCC research.

Although several nomograms have been explored to predict the prognosis of HCC and have been shown to be more accurate than traditional staging systems, they are limited to forward stepwise Cox regression risk factor screening, which is not conducive to multi-indicator model screening and small sample sizes (lasonos et al., 2008). Least absolute shrinkage and selection operator (LASSO) Cox regression, a method for variable selection and shrinkage in Cox proportional hazards model, constructs a penalty function to obtain a more refined model. It compresses the regression coefficients (the sum of the absolute values of the mandatory coefficients is less than a fixed value) to zero to achieve feature selection. Furthermore, LASSO Cox regression is more applicable in decisions with multiple clinical indicators, which not only extracts useful features effectively but also solves the problem of over-fitting (Tibshirani, 1997). In the present study, we aim to apply LASSO Cox regression to construct and validate a novel prognostic model by using the ATIRE-based risk score for predicting the probability of HCC prognosis.

## 2 Materials and methods

### 2.1 Data preparation and processing

**FIGURE 1:**
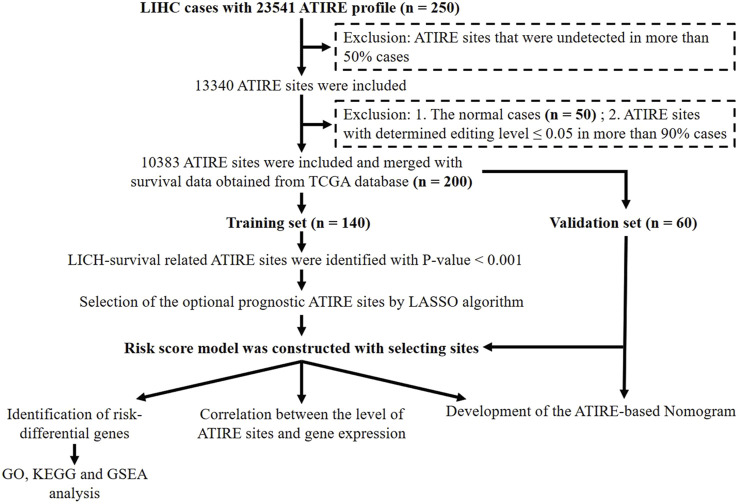
Flowchart of the identification of survival-related ATIRE sites and construction of the ATIRE-based nomogram for HCC.

In this study, the ATIRE profiles of TCGA-liver hepatocellular carcinoma (LIHC) were obtained from the Synapse website (https://www.synapse.org/#!Synapse:syn4382456) uploaded by Han et al. (2015). The corresponding clinical information and gene expression profiles of LIHC cases were downloaded from the TCGA database (https://portal.gdc.cancer.gov/). All these data are publicly available. The flowchart of data processing is presented in Figure 1. ATIRE sites were excluded when they were undetected in more than 50% cases, and the determined editing level was ≤0.05 in more than 90% cases. A total of 200 HCC patients and 50 adjacent normal cases from TCGA were evaluated, and 200 patients with 10,383 ATIRE sites were finally included. The patients were grouped into the training dataset (n = 140) and the validation dataset (n = 60) with a ratio of 7:3 using the R function “createDataPartition” to ensure that outcome events were distributed randomly between the two datasets (Wu et al., 2020).

### 2.2 Identification of LICH survival-related ATIRE sites and generation of the risk score model

In order to screen OS-related ATIRE sites, we first used the univariate Cox proportional hazards (Cox-PH) regression method by “survival,” “caret,” “glmnet,” and “survminer” R packages in the training dataset. A *p*-value < 0.001 was considered to be statistically significant. The Manhattan plot was applied to depict associations between ATIRE sites and LIHC survival. Then, the optional prognostic ATIRE sites were identified by the LASSO algorithm with the “glmnet” R package. LASSO–Cox regression was conducted in R packages to select prognostic ATIRE sites (Tibshirani, 1997). The ATIRE sites included in the model and the optimal value of the penalty coefficient were determined by running a 1,000-time 10-fold cross-validation probability. Then, coefficients of these RNA editing sites were extracted by Cox multivariate regression analysis, and the RNA editing levels were used to calculate the risk score, as shown in the following equation:

$$\text{Risk score}=\sum_{i}=Coefficient\;of\;\left(i\right)*RNA\;editing\;level\;\left(i\right).$$

Here, (i) represents the RNA editing site, and the coefficient of the RNA editing site is the regression coefficient of the RNA editing site, which represents the contribution of the RNA editing site to the prognostic risk score; the RNA editing level is calculated by dividing the number of reads where the RNA editing site occurs by the number of reads at the total coverage sites.

### 2.3 Survival analysis of the high-risk and low-risk groups

To reveal the prognostic value of the risk score model, survival analysis was performed using “survival” and “survminer” R packages. Using the median risk score as the cutoff point, we categorized the patients into low-risk and high-risk groups. In addition, patients were categorized into low-editing and high-editing groups according to the RNA editing level of these optional prognostic ATIRE sites. OS and progression-free survival (PFS) were calculated in the training dataset, validation dataset, and all samples. A *p*-value <  0.05 was considered to be statistically significant.

### 2.4 Construction and validation of the nomogram model based on the risk score

To explore the prognostic value of the ATIRE-based signature, a nomogram, including the risk score and the clinical features such as gender, age, grade, T stage, and M stage, was evaluated by multivariate Cox-PH regression with “rms” and “regplot” R packages. To assess the accuracy of the nomograms, we constructed a calibration diagram by the Cox regression predictive model. The X-axis and Y-axis represented predictive survival time and actual survival time, respectively. The concordance index (C-index) was employed to observe the discrimination between the predicted probability and the actual outcome. Generally, C-index ≥0.7 was considered as a strong predictive ability. Meanwhile, time-dependent ROC curves with “timeROC” R packages were applied to validate the sensitivity and specificity of the nomogram (Rousson and Zumbrunn, 2011). Calibration curves were performed to predict the OS at 1, 2, and 3 years. In addition, decision curve analysis (DCA) with “ggDCA” R packages was used to evaluate the clinical utility of the nomogram.

### 2.5 Effect of the selected ATIRE sites on target genes’ expression

To explore the possible effect of the selected ATIRE sites on the expression of target genes, Spearman correlation analysis, typically used when data did not follow a normal distribution, was carried out to compute their correlation coefficient in the high-risk group.

### 2.6 Identification of risk-differential genes and functional enrichment

To further demonstrate the potential effect of the ATIRE risk score on the transcriptome expression, we compared the gene expression differences between the high- and low-risk groups with “limma” R package (Ritchie et al., 2015). |log2FC| >1 and *p* < 0.05 were defined as the cutoff values to identify valuable signaling pathways. In order to analyze the risk-differential genes for functional enrichment, “clusterProfiler,” “org.Hs.eg.db,” and “enrichplot” were employed to perform the Gene Ontology (GO) clustering analysis and Kyoto Encyclopedia of Genes and Genomes (KEGG) pathway analysis. Only pathways with a *p*-value < 0.05 were eligible for enriched biological processes. Furthermore, gene set enrichment analysis (GSEA), a pathway enrichment method, was performed to identify intergroup pathway differences through enrichment scoring.

### 2.7 Statistical analysis

All statistical analyses were executed by using R software (version 4.2.1). In addition to those methods already mentioned, the difference of demographic and clinicopathological features between the training dataset and the validation dataset was tested using the chi-squared test. Unless otherwise stated, a *p*-value <0.05 was regarded to be statistically significant.

## 3 Results

### 3.1 Identification of HCC survival-related ATIRE sites and generation of the ATIRE risk score

**FIGURE 2:**
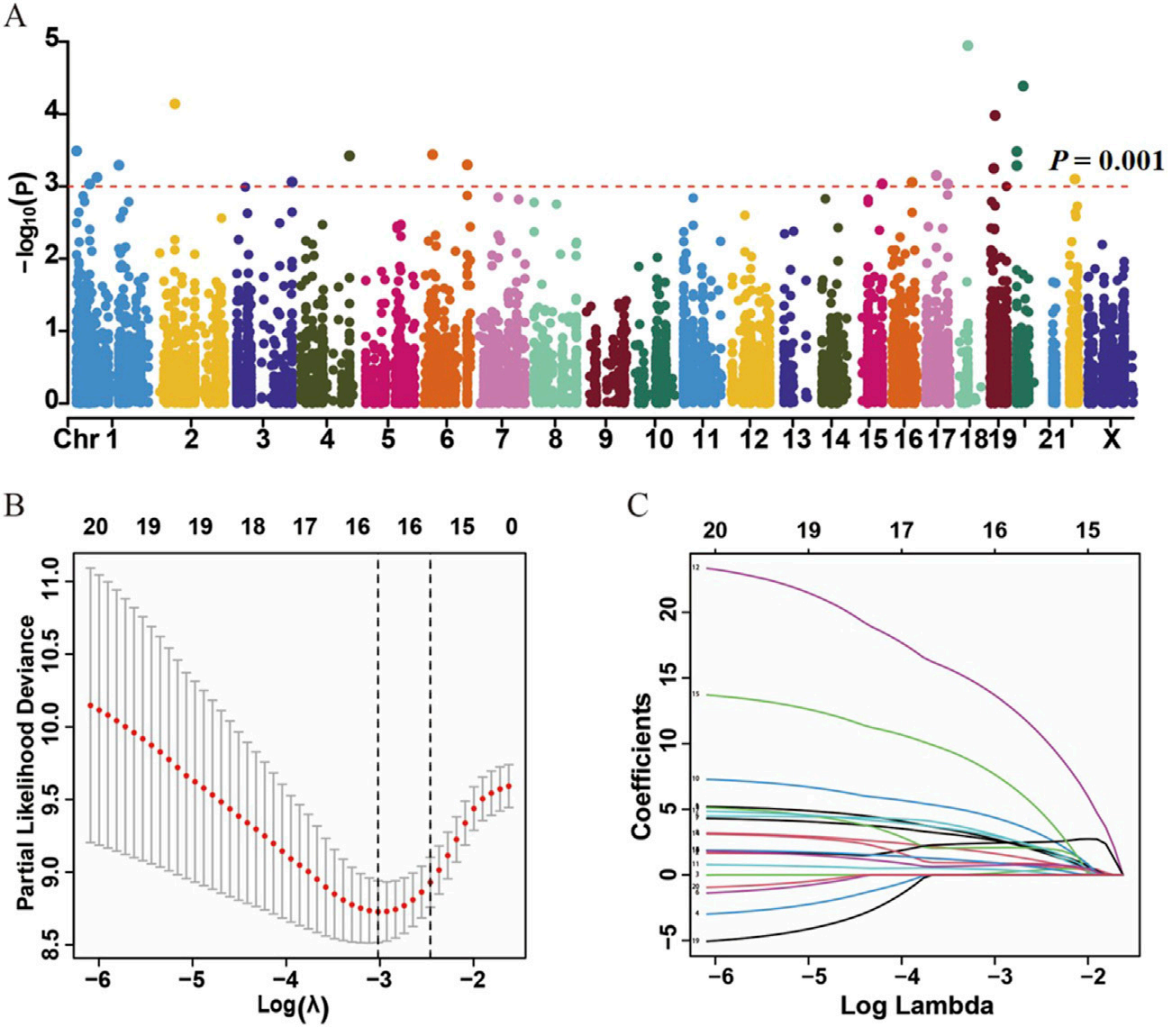
Identification of ATIRE prognostic sites for HCC patients. **(A)** Manhattan plot depicts the association between ATIRE sites and HCC survival. P-values in –log10 scale calculated from the univariate Cox-PH model as the X-axis and the chromosomal location of the ATIRE sites as the Y-axis. The dotted line indicates the cutoff of significance with the *p-*value as 0.001. **(B)** Selection of optimal ATIRE sites (lambda) and dotted vertical lines by the LASSO regression analysis. **(C)** LASSO coefficient profiles in the LASSO regression model.

A total of 200 HCC patients were included in the present study based on the inclusion criteria, and their baseline characteristics are summarized in Table 1. There were no significant differences between the training and validation datasets in terms of age, gender, grade, and tumor stage, indicating that the grouping was reasonable and unbiased. The Manhattan plot depicting ATIRE sites with *p* < 0.001 in the Cox-PH model was utilized to test associations between 10,383 ATIRE sites and HCC survival in the training dataset (Figure 2A). Subsequently, we selected TMEM192 (chr4:165996838A > I), MRI1 (chr19:13883596A > I), LRCH3 (chr3:197612267A > I), IQCG (chr3:197612267A > I), APOL6 (chr22:36057355A > I), IPP (chr1:46160965A > I), ZNF397 (chr18:32829377A > I), C2 (chr6:31902666A > I), CTH (chr1:70910693A > I), PTGER3 (chr1:70910693A > I), and TRIM65 (chr17:73885988A > I) as the optimal prognostic sites by the LASSO analysis to generate the ATIRE risk score (Figures 2B,C). By using X-Tile (Camp et al., 2004), the editing levels of the above ATIRE sites were grouped into “low” and “high,” and the HRs for associations between these sites and HCC OS are shown in the S1 DataSheet. In addition, we also analyzed survival probabilities for the nine selected ATIRE sites (*p* < 0.001) in the total patients (Supplementary Figure S1).

**TABLE 1:** Frequency distribution of clinicopathological characteristics of LIHC patients.

Characteristics	Total (n = 200)	Training dataset (n = 140)	Validation dataset (n = 60)	*P*-value
Age (years), n (%)				
≤65	116 (58.00)	79 (56.43)	37 (61.67)	0.60
>65	84 (42.00)	61 (43.57)	23 (38.33)	
Gender, n (%)				
Female	70 (35.00)	55 (39.29)	15 (25.00)	0.08
Male	130 (65.00)	85 (60.71)	45 (75.00)	
Grade, n (%)				
G1–2	133 (66.50)	93 (66.43)	40 (66.67)	1.00
G3–4	62 (31.00)	44 (31.43)	18 (30.00)	
Unknown	5 (2.50)	3 (2.14)	2 (3.33)	
Stages, n (%)				
I–II	124 (62.00)	89 (63.57)	35 (58.33)	0.29
III–IV	62 (31.00)	39 (27.86)	23 (38.33)	
Unknown	14 (7.00)	12 (8.57)	2 (3.33)	
T stages, n (%)				
1–2	134 (67.00)	97 (69.29)	37 (61.67)	0.30
3–4	64 (32.00)	41 (29.29)	23 (38.33)	
Unknown	2 (1.00)	2 (1.43)	0 (0.00)	
M stages, n (%)				
0	145 (72.50)	103 (73.57)	42 (70.00)	0.48
1	4 (2.00)	4 (2.86)	0 (0.00)	
Unknown	51 (25.50)	33 (23.57)	18 (30.00)	
N stages, n (%)				
0	125 (62.50)	84 (60.00)	41 (68.33)	1.00
	3 (1.50)	2 (1.43)	1 (1.67)	
Unknown	72 (36.00)	54 (38.57)	18 (30.00)	

*P*-values were calculated by the chi-square test.

**FIGURE 3:**
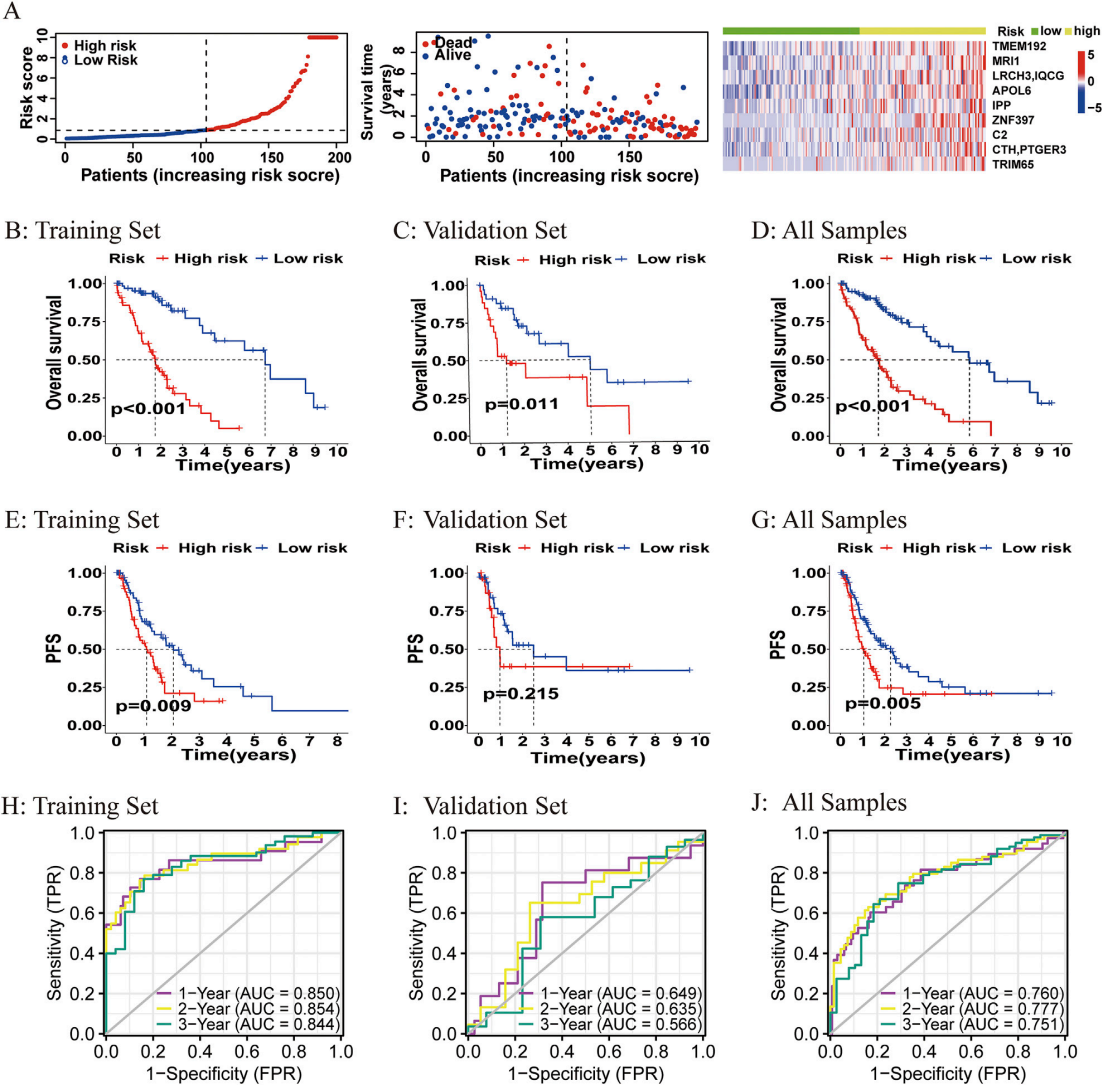
Correlations between the ATIRE risk score and prognosis of HCC patients. **(A–G)** Distribution of the ATIRE risk score, survival status, and editing levels of the nine ATIRE sites in overall HCC patients **(A)**, and Kaplan–Meier plots to visualize the OS and PFS in the training set **(B, E)**, validation set **(C, F)**, and combined set **(D, G)**. **(H–J)** Receiver operating characteristic (ROC) curve analysis for the prognostic value of the prognostic model for different years in the training set **(H)**, validation set **(I)**, and combined set **(J)**. Data from TCGA (median risk score as the cutoff value). AUC, area under the curve.

The ATIRE risk score was generated by the sum of coefficients of each ATIRE site from the LASSO analysis. The distribution of the risk score, survival status, and the editing levels of the ATIRE sites were observed in the overall HCC patients (Figure 3A) and in both the training and validation datasets (Supplementary Figure S2). Patients with low-risk scores had a higher OS than those with high-risk scores in both the training dataset (*p* < 0.001) and the validation dataset (*p* = 0.011), as well as in the combined dataset (*p* < 0.001; Figures 3B–D). Significant associations between the ATIRE risk score and PFS were also observed in both the training dataset (*p* = 0.009) (Figure 3E) and the combined dataset (*p* = 0.005) (Figure 3G). However, the difference of PFS between high-risk scores and low-risk scores did not reach statistical significance in the validation dataset (Figure 3F), which might be due to the limited sample size. Then, we constructed an ROC curve and showed the AUC values of 1-, 2-, and 3-year OS in the training dataset, validation dataset, and the combined dataset (Figures 3H–J).

### 3.2 Construction of the ATIRE-based nomogram and evaluation of predictive performance on HCC survival

**FIGURE 4:**
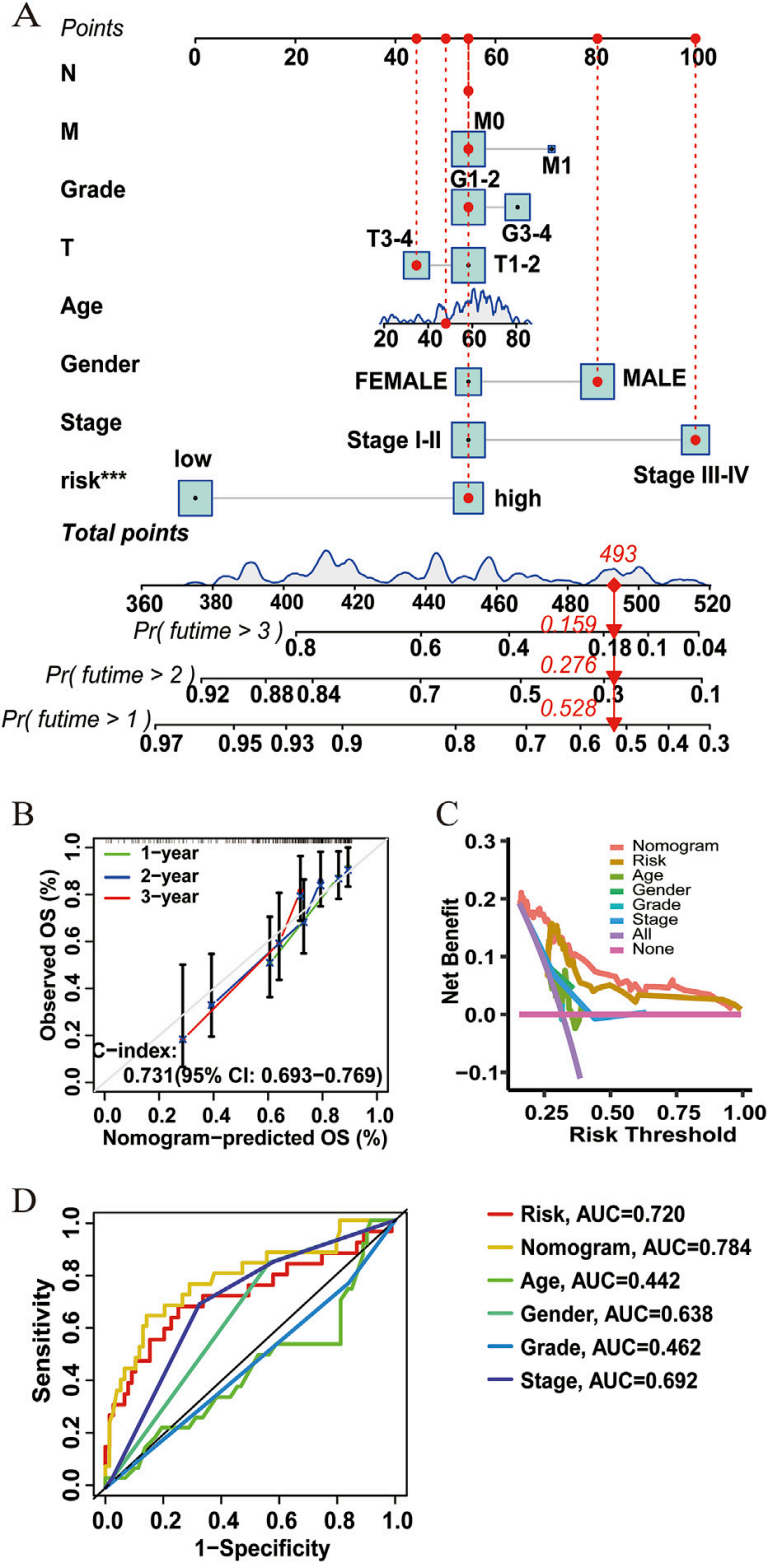
Performance of the prognostic nomogram based on the ATIRE risk score and clinicopathological features. **(A)** Nomogram for predicting 1-, 2-, and 3-year OS probability of HCC patients. **(B)** Calibration curves for predicting patients’ OS at 1, 2, and 3 years in overall HCC patients. **(C)** Decision curves depict the comparison in net benefits for predicting 1-year OS rate of different nomograms that include single ATIRE risk score, age, gender, grade, stage, and their combination. **(D)** ROC curves verify the prognostic performance of different nomograms including the single ATIRE risk score, age, gender, grade, stage, and their combination.

A nomogram for predicting HCC survival was established with the ATIRE risk score and several risk factors, including the N stage, M stage, T stage, gender, and age at diagnosis (Figure 4A). As shown in Figure 4B, the calibration plots demonstrated a resemblance in all cohorts between the observed OS rate and nomogram-predicted OS rate at 1, 2, and 3 years. The Harrell’s C-indexes were 0.731 (95% CI = 0.693–0.769) in the overall HCC patients. Furthermore, the decision curve and ROC curve indicated that the nomogram with the ATIRE risk score and several risk factors, including age, gender, and grade, had a better net benefit and prognostic performance than the nomogram with single ATIRE risk score or risk factors (Figures 4C,D).

### 3.3 Effects of the editing levels of ATIRE sites on the expression of host genes

**FIGURE 5:**
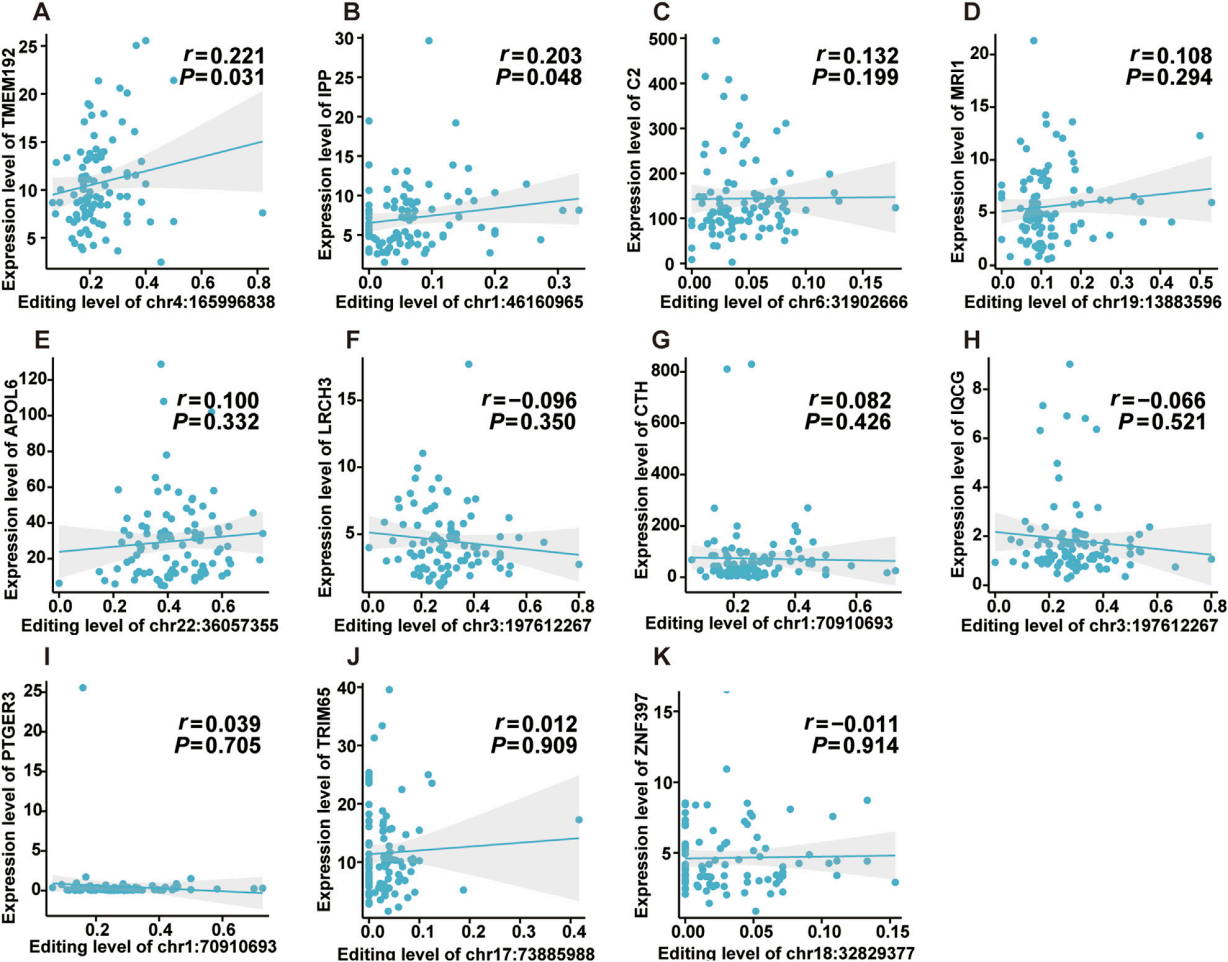
Effects of editing levels of ATIRE sites on the expression of host genes. **(A–K)** Correlations between editing levels of the nine selected ATIRE sites and expressions of host genes. P and r were calculated by the Spearman correlation test.

Previous studies reported that ATIRE was involved in physiological and pathological processes by mediating host gene expression (Licht et al., 2019; Kapoor et al., 2020). Thus, we investigated the effects of the editing levels of ATIRE on host gene expression. As shown in Figures 5A,B, there were significantly positive associations between the editing level of chr4:165996838A > I and TMEM192 (*P* = 0.031), as well as the editing level of chr1:46160965A > I and IPP (*P* = 0.048). Nevertheless, no significant association was observed for the following ATIRE sites and their related host genes: the chr19:13883596A > I and MRI1, chr3:197612267A > I and LRCH3 or IQCG, chr22:36057355A > I and APOL6, chr18:32829377A > I and ZNF397, chr6:31902666A > I and C2, chr1:70910693A > I and CTH or PTGER3, and chr17:73885988A > I and TRIM65 (Figures 5C–K).

### 3.4 Differentially expressed genes and related biological pathways associated with the ATIRE risk score

**FIGURE 6:**
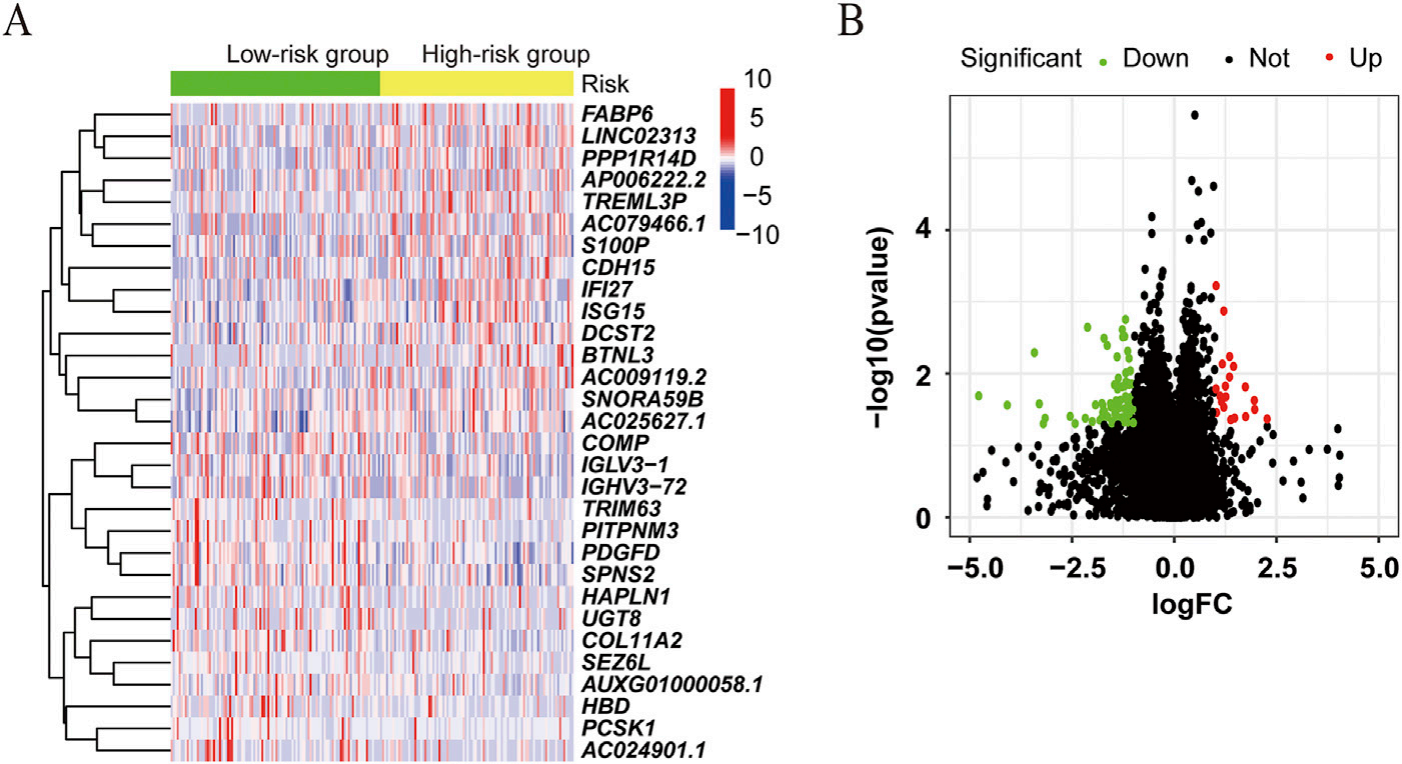
Differentially expressed genes associated with the ATIRE risk score. **(A)** Differently expressed genes were presented by volcano plot in the high-risk group versus the low-risk group. **(B)** Heatmap of gene expression revealed the top 15 upregulated and downregulated genes with significant differences.

**FIGURE 7:**
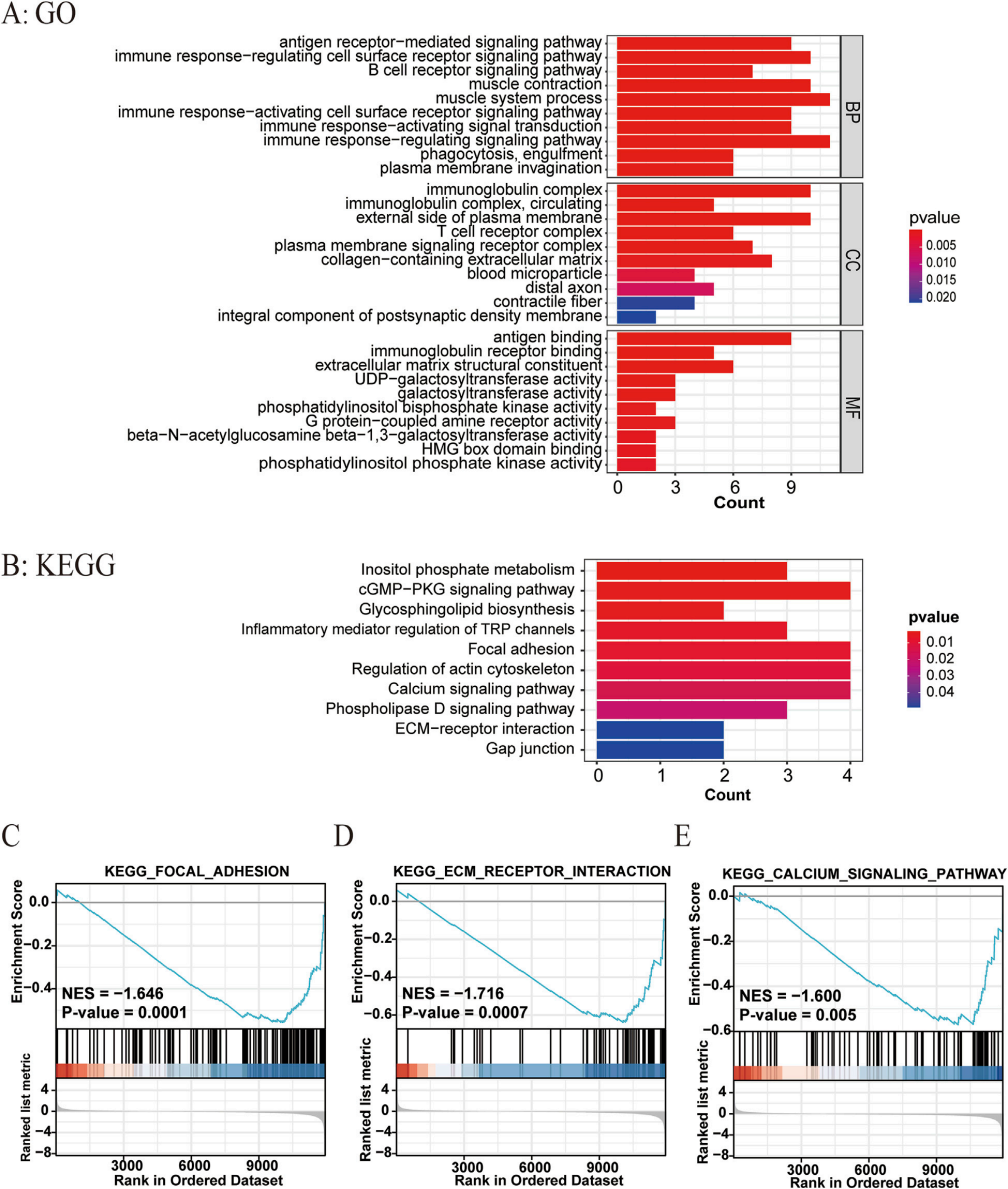
Related biological pathways associated with the ATIRE risk score. **(A, B)** GO and KEGG enrichment analyses for ATIRE-related genes. **(C–E)** GSEA enrichment plot of hallmarks in the high-risk group versus the low-risk group.

We further compared the gene expression difference in patients with high and low ATIRE risk scores. Differently expressed genes were presented by volcano plot, and heatmap of gene expression revealed the top 15 upregulated and downregulated genes with significant differences between the two groups (Figures 6A,B). GO and KEGG analyses showed that these genes were enriched in several pathways, such as the cGMP–PKG signaling pathway, focal adhesion, regulation of actin cytoskeleton, ECM–receptor interaction, and calcium signaling pathway (Figures 7A,B). Consistently, GSEA enrichment analysis found that target genes were involved in focal adhesion, ECM–receptor interaction, and calcium signaling pathway (Figures 7C–E).

## 4 Discussion

Given that the majority of patients who are diagnosed with HCC are at an advanced stage, the overall prognosis is poor for patients with HCC (Llovet et al., 2021). For the past few years, despite substantial progress in the treatment of HCC, the management of this disease remains complicated (Yang and Heimbach, 2020).

ATIRE, a typical RNA-editing manner, was found to be implicated in tumorigenesis by participating in several biological processes. Although increasing evidence disclosed that several ATIRE events were involved in various cancer survival patients (Gumireddy et al., 2016; Shigeyasu et al., 2018; Xu et al., 2019), there was lack of novel prognostic signatures based on the ATIRE risk score for predicting the survival of patients with HCC. In this study, we, for the first time, developed a nomogram based on the ATIRE risk score and found its prognosis value on the OS and PFS of HCC.

Through analysis of TCGA–HCC ATIRE profiling with Cox-PH regression and LASSO algorithm, nine ATIRE sites were screened as optimal prognostic factors to establish a nomogram for predicting the OS of HCC. Overall, the constructed nomogram displayed a good performance and stability on predicting HCC OS (1-year) with Harrell’s C-indexes as 0.850, 0.649, and 0.760 in the training dataset, validation dataset, and the combined dataset, respectively. More importantly, the predictive efficiencies were also verified by the calibration plots. Consequently, the constructed nomogram may help clinicians in the better diagnosis and management of patients with HCC.

For the selected sites, most of them have been reported to be implicated in cancer genesis and development. For instance, deficiency of *TMEM192* in hepatoma HepG2 cells resulted in increased apoptosis and growth inhibition by the mitochondrial pathway through autophagy (Liu et al., 2012). APOL6, a novel proapoptotic Bcl-2 homology 3-only protein, induced mitochondria-mediated apoptosis in p53-null colorectal cancer cells (Liu et al., 2005). The expression of IPP was significantly increased in the human breast tumor samples compared with the non-cancer tissues (Govindaraj et al., 2012). The single-nucleotide polymorphism of complement component 2 (C2) was reported to be significantly associated with HCC (Clifford et al., 2010). Notably, a recent study demonstrated the correlation of C2 with immune infiltration in HCC and underlined that C2 was an independent factor for the prognosis of HCC (Ning et al., 2020). Cystathionine-gamma-lyase (CTH) was a pivotal enzyme of the transculturation pathway. CTH-expressing T cells reduced glycine, serine, and proline concentrations in the tumor interstitial fluid and had a superior control of tumor growth (Lancien et al., 2021). Additionally, bioinformatics analysis showed that CTH was downregulated in HCC and was correlated with a poor prognosis for HCC patients (Cai et al., 2020). PTGER3 induced ovary tumorigenesis and conferred resistance to cisplatin therapy through the Ras–MAPK/ERK–ETS1–ELK1/CFTR1 axis (Rodriguez-Aguayo et al., 2019). Moreover, as an oncogene, *TRIM65* promoted cell growth and tumor metastasis in HCC *via* ubiquitylation of Axin1 to activate the β-catenin signaling pathway (Yang et al., 2017). Although these genes have been reported to be involved in the development and progression of cancer, further experimental validation was needed to elucidate how ATIRE sites affect the expression of these genes and, thus, cancer prognosis.

Presently, the underlying mechanisms remained unclear on how ATIRE sites were associated with HCC survival. A previous study demonstrated that ATIRE of *AZIN1* promoted cell proliferation through the neutralization of antizyme-mediated degradation of ornithine decarboxylase and cyclin D1 in HCC (Chen et al., 2013). Hu et al. (2015) also showed that RNA over-editing of *BLCAP* promoted cell proliferation *via* activating the AKT/mTOR signal pathway. In addition, accumulating evidence indicated that ATIRE could contribute to amino acid changes, alternative splicing, microRNA-mRNA redirection, or RNA-binding protein-mRNA redirection (Xu et al., 2018; Chan et al., 2020). Recently, Zhang et al. (2024) detailed the mechanisms by which ATIRE was involved in different types of tumors, including alterations in the noncoding regions of mRNAs that affected the immunogenicity of double-stranded RNAs, modifications in the coding regions of mRNAs that cause non-synonymous amino acid mutations, changes to the structure of circRNAs, and effects on miRNA maturation. These ATIRE events result in the activation or inactivation of oncogenic pathways, ultimately promoting the development of tumors. In our study, we observed significantly positive correlations of chr4:165996838A > I level with TMEM192 and chr1:46160965A > I level with IPP, which suggested a posttranscriptional effect of these ATIRE sites in HCC on host genes’ expression. In addition, most of the selected ATIRE sites were located in the 3′-UTR of host genes. It was plausible that they mediated host genes’ expression by interfering with the binding ability of microRNAs or RNA-binding proteins. Recently, single-cell multi-omics technologies had provided novel insights into the molecular features and biological processes, and integrative analysis of single-cell multi-omics data enhanced our understanding of the regulatory mechanisms of the complex tumor (Song et al., 2022). Further single-cell multi-omics data integration combined with molecular biological experiments is needed to elucidate the elaborated mechanisms underlying the associations between the ATIRE sites and HCC.

Inevitably, there were several limitations in the current study. First, there was selection bias during the process of data preparation and processing, which might affect the authenticity of the association. Second, we only performed the analysis using the TCGA data and lacked an external validation in other databases, which might undermine the reliability of the ATIRE-based model. Finally, there was lack of detailed therapeutic options in the TCGA database, which hampered us from confirming the effect of the ATIRE-based model on the application of different treatment strategies. Therefore, future prospective studies are needed with multicenter, larger, and more detailed information to validate these results.

## 5 Conclusion

In summary, we constructed and validated a prognostic signature based on the ATIRE risk score, which could serve as a potential tool for HCC survival prediction and provide new insights into the diagnosis and treatment of HCC.

## Data Availability

Publicly available datasets were analyzed in this study. This data can be found here: https://portal.gdc.cancer.gov/.

## Supplementary material

SUPPLEMENTARY DATA SHEET S1 The HRs for associations between the ATIRE sites and OS of HCC patients.

SUPPLEMENTARY FIGURE S1 Survival probabilities for the nine selected ATIRE sites in the overall HCC patients.

SUPPLEMENTARY FIGURE S2 The distribution of risk score, survival status, and the editing levels of the ATIRE sites in both the training and validation sets.

## Abbreviations

ATIRE, adenosine-to-inosine RNA editing; HCC, hepatocellular carcinoma; TCGA, The Cancer Genome Atlas; OS, overall survival; LIHC, liver hepatocellular carcinoma; TNM, tumor node metastasis; Cox-PH, Cox proportional hazards; ROC, receiver operating characteristic; ADAR, adenosine deaminase acting on RNA; AZIN1, antizyme inhibitor 1; BLCAP, bladder cancer-associated protein; LASSO, least absolute shrinkage and selection operator; PFS, progression-free survival; C-index, concordance index; DCA, decision curve analysis; GO, Gene Ontology; KEGG, Kyoto Encyclopedia of Genes and Genomes; GSEA, gene set enrichment analysis.
