# Supplementary figures and images for: A novel adenosine-to-inosine RNA editing-based nomogram for predicting prognosis of hepatocellular carcinoma
Source: Front Pharmacol. 2025 May 14;16:1547320. doi: 10.3389/fphar.2025.1547320 (PMC12116476; doi:10.3389/fphar.2025.1547320)

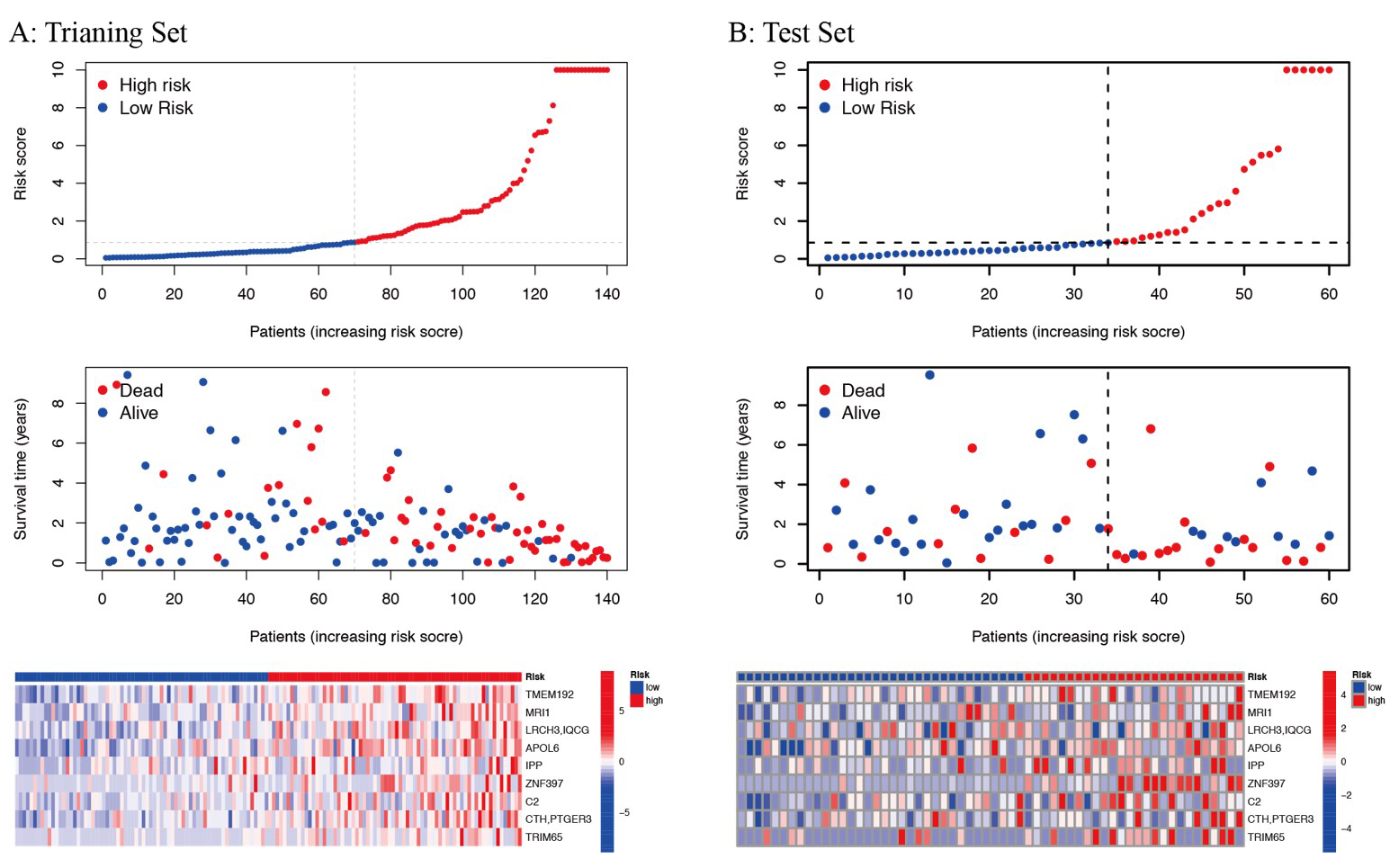

Supplement: Supplementary file 1 [file Image2.tif]

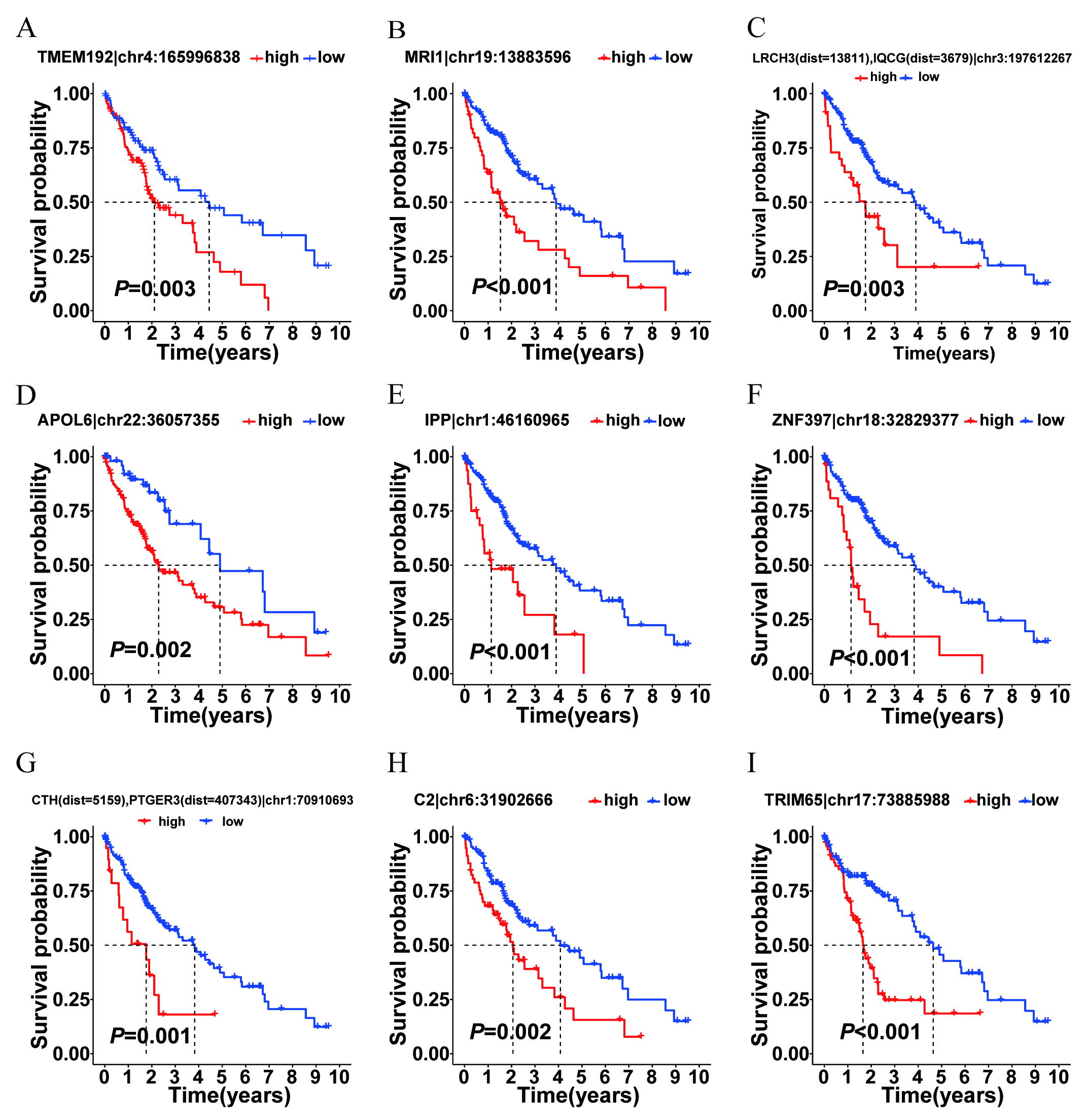

Supplement: Supplementary file 2 [file Image1.tif]
